# Effectiveness of Mobile Applications Running on Smartphones to Promote Physical Activity: A Systematic Review with Meta-Analysis

**DOI:** 10.3390/ijerph17072251

**Published:** 2020-03-27

**Authors:** Anabela G. Silva, Patrícia Simões, Alexandra Queirós, Nelson P Rocha, Mário Rodrigues

**Affiliations:** 1 CINTESIS.UA, School of Health Sciences, University of Aveiro, Campus Universitário de Santiago, 3810-193 Aveiro, Portugal; 2 School of Health Sciences, University of Aveiro, Campus Universitário de Santiago, 3810-193 Aveiro, Portugal; patricianunessimoes@ua.pt; 3 IEETA, School of Health Sciences, University of Aveiro, Campus Universitário de Santiago, 3810-193 Aveiro, Portugal; alexandra@ua.pt; 4 IEETA, Department of Medical Sciences, University of Aveiro, Campus Universitário de Santiago, 3810-193 Aveiro, Portugal; npr@ua.pt; 5 IEETA, Higher School of Technology and Management of Águeda, University of Aveiro, R. Cmte, Pinho e Freitas 5, 3750-127 Águeda, Portugal; mjfr@ua.pt

**Keywords:** mobile applications, physical activity, self-efficacy, sedentarism

## Abstract

Mobile applications reach a high number of individuals at low costs. This systematic review investigated the effectiveness of mobile application-based interventions to increase physical activity (PA) and self-efficacy and to decrease sedentarism. Randomized controlled trials (RCTs) and quasi-RCTs investigating the effect of PA interventions using an app compared to no intervention or traditional interventions were included. Pooled effects using the standardized mean difference (SMD) or the weighted mean difference (WMD) were calculated and the overall quality of the evidence was rated using the GRADE. Eleven studies were included. In the short term, pooled estimates showed a small and positive effect in the number of steps favoring interventions using a mobile app when compared with no interventions (WMD = 1579.04, 95%CI 454.04 to 2703.38) and with traditional interventions (WMD = 665.96, 95%CI 167.92 to 1164.00). For self-efficacy and at follow-up, results favoured traditional interventions (WMD = −8.20, 95%CI −14.25 to −2.15). Non-significant results were found for the remaining comparisons. The quality of the evidence ranged from very low to low. There is very low to low quality evidence that interventions using mobile apps running on smartphones, when combined with traditional interventions, are superior to traditional interventions in the short term. Further high-quality studies are required.

## 1. Introduction

Physical activity is associated with countless health benefits, independently of age. For children and adolescents, in particular, physical activity is associated with positive changes in adiposity, skeletal health, psychological health, improved self-esteem, fewer depressive symptoms [1] and improved cardiorespiratory fitness [2]. For those aged 18 to 85 years old, physical activity is associated with the prevention of obesity, coronary heart disease, type 2 diabetes mellitus or dementia. For older adults, physical activity has been associated with reduced health care use [3,4], the better management of chronic conditions, delayed decline in function and increased longevity [5,6,7]. Therefore, promoting physical activity is of primordial relevance for all health professionals, independently of care setting and level of care (primary, secondary or tertiary).

Mobile applications (apps) running on mobile devices, such as smartphones, have been used to promote physical activity. One of their main advantages is that their use is widely disseminated among the general population. There were around 1.86 billion smartphones in 2015 and this number is expected to increase to 2.87 billion in 2020 [8]. Similarly, the number of apps related to health and fitness in the Google Play and App Store was around 102.962 and 97.844 in June 2018, respectively [9,10,11]. Therefore, apps running on smartphones show a huge potential for the promotion of physical activity, as they seem to offer the possibility of reaching a large number of individuals at reduced costs, are virtually always accessible and allow for real-time monitoring and feedback, eventually facilitating individually-tailored interventions [12,13]. However, systematic reviews investigating the effectiveness of apps running on smartphones to promote physical activity are lacking. Therefore, this manuscript presents a systematic review with meta-analysis to investigate the effectiveness of using mobile app-based interventions to increase physical activity levels, compared to no intervention or interventions that do not use mobile apps in adults aged 18 to 65 years old, with or without pathology. The secondary aims were to investigate the effectiveness of mobile apps to decrease sedentarism and increase self-efficacy, as the first seems to modulate the effects of physical activity [14] and the second seems to impact adherence to physical activity [15,16].

## 2. Materials and Methods 

This manuscript is in line with the Preferred Reporting Items for Systematic Reviews and Meta-Analyses guidelines for conducting a systematic review [17].

### 2.1. Literature Search

Pubmed, Science Direct, Web of Science, Physiotherapy Evidence Database (PEDro), Academic Search Complete and IEEE Xplore were searched for relevant articles on the effectiveness of mobile apps assessing aspects of physical activity. All databases were searched since 1 January 2000, as it was around this year that faster ICT applications and services have begun to be available, with the appearance of three-generation cellular systems [18]. Combinations of the following search terms were used: “physical activity”, “physical fitness”, “walk*”, “pedometer”, “mobile application”, “mobile app”, “app”, “smartphone application” and “smartphone app”. The search was performed on the 29 and 30 October 2017 without language restrictions. PubMed was searched using MeSH terms. The reference lists of included articles were also screened for reports not identified through electronic searches. We did not use sports-related search terms, as physical activity is a broader term that encompasses the latter. 

### 2.2. Eligibility Criteria

Type of studies: randomized controlled trials (RCTs) or quasi-experimental trials (quasi-RCTs) that used a mobile application to promote physical activity. Only studies with full-text available and in English or Portuguese were included. Articles without an author and/or without enough detail to allow the characterization of study procedures were excluded.

Participants: studies including human participants from both genders with or without any pathology and aged 18 to 65 years old. 

Intervention and comparators: a physical-activity based intervention delivered using a mobile app, both as a standalone treatment, or combined with other treatments and compared against a group receiving another type of intervention, a sham-intervention or no intervention at all. For this review, a mobile app was defined as: “A software/set of program that runs on a mobile device and performs certain tasks for the user” [19]. Studies that used apps that did not primarily target physical activity were excluded.

Not meeting one inclusion criteria was sufficiente for exclusion. When studies did not meet more that one criteria, we listed the first identified reason for exclusion.

Outcomes: the main outcome of interest was a change in physical activity, defined as any movement of the body produced by the skeletal muscle that results in energy expenditure, which can be objectively characterized by measuring body displacement or energy expenditure [20,21]. Therefore, steps, distance, time spent performing an activity, energy expenditure or self-reported physical activity were accepted as indicators of the primary outcome of interest. Also, sedentarism (measured for example, as time spent seated) and self-efficacy (measured through self-reported questionnaires) were included if reported (secondary outcomes).

### 2.3. Selection of Studies and Data Extraction

Search results were handled with Mendeley Desktop version 1.18 (Elsevier, London, United Kingdom). Titles, abstracts and, subsequently, full-text articles were independently screened by two reviewers (AGS and PS), against the inclusion and exclusion criteria. Disagreements were solved by discussion, until consensus was reached. One reviewer (PS) extracted all relevant information from included studies to a standardized form in Microsoft Excel 2016 (Microsoft Corporation, Redmond, Washington) and a second reviewer (AGS) checked it for accuracy. Retrieved information included author, year of publication, study design, name of the mobile application, number and characteristics of the participants, description of the intervention in the control and experimental groups, length of follow-up, types of outcomes and results. 

### 2.4. Risk of Bias and Quality Assessment in the Included Studies

Two reviewers (AGS and PS) independently assessed quality and risk of bias of included studies using the PEDro scale and the Cochrane Collaboration’s risk of bias tool. The PEDro scale is based on the list developed by Verhagen et al. [22]., using the Delphi method, and is constituted by 11 items: eligibility criteria, random allocation, concealed allocation, similarity at baseline, subject blinding, therapist blinding, assessor blinding, follow-up for at least 85% of subjects, intention-to-treat analysis, between-group statistical comparison and point, and variability measures. Of the 11 items, only 10 items contribute to the total PEDro score. The 10 items are scored as either present (1) or absent (0). The total score is obtained through the sum of the points assigned to each item [23]. The quality of included studies was considered high when the total score was 6 [23]. The Cochrane Collaboration’s risk of bias tool is constituted by the following domains: selection bias (sequence generation and allocation concealment), performance bias (blinding of participants and personnel), detection bias (blinding of outcomes assessment), attrition bias (incomplete outcome data), reporting bias (selective reporting) and other bias. Each domain was assessed as “low risk”, “high risk”, or as an “unclear risk” [24]. Disagreements between the two reviewers (PS and AGS) were solved by discussion until a consensus was reached. 

### 2.5. Data Synthesis and Analysis

For each outcome, mean (final or change from baseline) and standard deviations (SD) and sample size of control and experimental groups were retrieved from the included studies. When data were missing, we contacted the authors of the study, used the information presented in graphical format or calculated data with available information using the recommendations of the Cochrane Handbook for Systematic Reviews of Interventions [24]. The effect measures used were weighted mean difference (WMD) and 95% confidence intervals (CI) for continuous data using a random-effects model according to Cochrane recommendations. WMD was used as a summary statistic when outcome measurements in all studies were made on the same scale and SMD was used when all studies assess the same outcome, but measure it using different metrics, as recommended [24]. SMD was calculated using the following formula: SMD = (Difference in mean outcome between groups)/(pooled standard deviation) [24]. SMDs of 0.2, 0.5, and 0.8 were rated as small, moderate, and large, respectively [25]. For studies included in the meta-analysis, heterogeneity between studies was assessed using the Cochran Q test (also known as Chi^2^ test) [26] and the Higgins I^2^ [27]. For the Chi^2^ test, it was determined that a *p*-value ≤ 0.10 indicates statistically significant heterogeneity. The Higgins I^2^ indicates the magnitude of heterogeneity and was interpreted as I^2^= 0% to 24%: low heterogeneity; I^2^ = 25% to 49%: moderate heterogeneity; I^2^= 50% to 74%: substantial heterogeneity; and I^2^ = 75% to 100%: considerable heterogeneity [27]. A sub-group analysis was performed for studies giving post-intervention mean differences and studies giving mean change from baseline. [24] No sensitivity analysis was performed. Meta-analyses were conducted using the MetaXL version 5.3 for Microsoft Excel (http://www.epigear.com). 

### 2.6. Risk of Bias Across Studies

The overall quality of the evidence for each outcome was assessed using the Grading of Recommendations Assessment, Development and Evaluation (GRADE) system [28], independently by two authors (AGS and PS) and the final decision was reached through discussion and consensus. The decision to downgrade or not the evidence considered four criteria: (i) study design and risk of bias—downgraded when more than 25% of the studies were scored with high risk of bias (PEDro score lower than 6 and qualitative analysis of the risk of bias tool); (ii) inconsistency—downgraded when substantial heterogeneity was presented with I^2^ > 50%; (iii) indirectness—downgraded when studies were from heterogeneous samples (e.g., asymptomatic vs. with a disease) and/or there was a co-intervention in the experimental group (e.g., app plus face-to-face intervention); (iv) imprecision—downgraded if less than 400 participants were included in the meta-analysis. Publication bias was not assessed. Evidence from RCTs was downgraded from high quality by one level for each factor that we considered to be present. Observational studies (quasi-RCTs) were upgraded from low quality by one level, if studies showed large effects and there was no obvious bias explaining those effects [29].

## 3. Results

### 3.1. Study Selection

**Figure 1:**
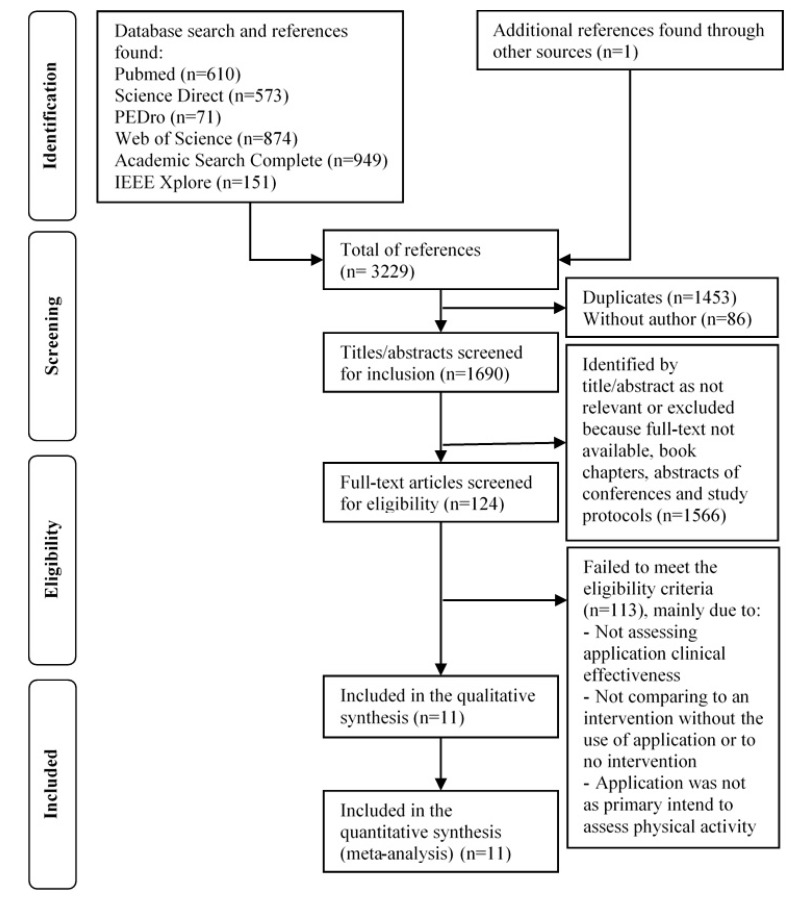
Preferred Reporting Items for Systematic Reviews and Meta-analysis (PRISMA) flow diagram. Abbreviations: PEDro, Physiotherapy Evidence Database.

The literature search identified 3229 references, of which 124 full-text articles were retrieved and read for eligibility after the selection process described in Figure 1. From these, 113 did not meet the inclusion criteria. Reasons for exclusion were (counting only the first reason identified): articles did not assess effectiveness (n = 62), did not compare a mobile app intervention with other interventions/no intervention (n = 20), did not primarily measure physical activity (n = 15), did not evaluate a mobile app (n = 9), did not measure physical activity (n = 5), did not have at least one post-intervention measurement (n = 1) and did not assess the effectiveness in adults (n = 1). A total of 11 studies were included in this review.

### 3.2. Description of the Included Studies

The studies included in this review were published between 2014 and 2017. The number of articles published per year was: one article in 2014, one article in 2015, five articles in 2016 and four articles in 2017. There were two quasi-RCTs and nine RCTs. The sample size of included studies ranged between 23 and 356 participants. Of the 11 studies included in the review, 8 used participants with chronic diseases such as chronic obstructive pulmonary disease (COPD), diabetes type 2, obesity, stroke and breast cancer [30,31,32,33,34,35,36,37]. Of the four remaining studies, one study used healthy participants [38], one used sedentary pregnant women [39], and one did not provide details on the type of participants used [40]. A detailed characterization of included studies is presented in Supplementary Table S1. 

### 3.3. Risk of Bias of Included Studies and Quality of Evidence

**Figure 2:**
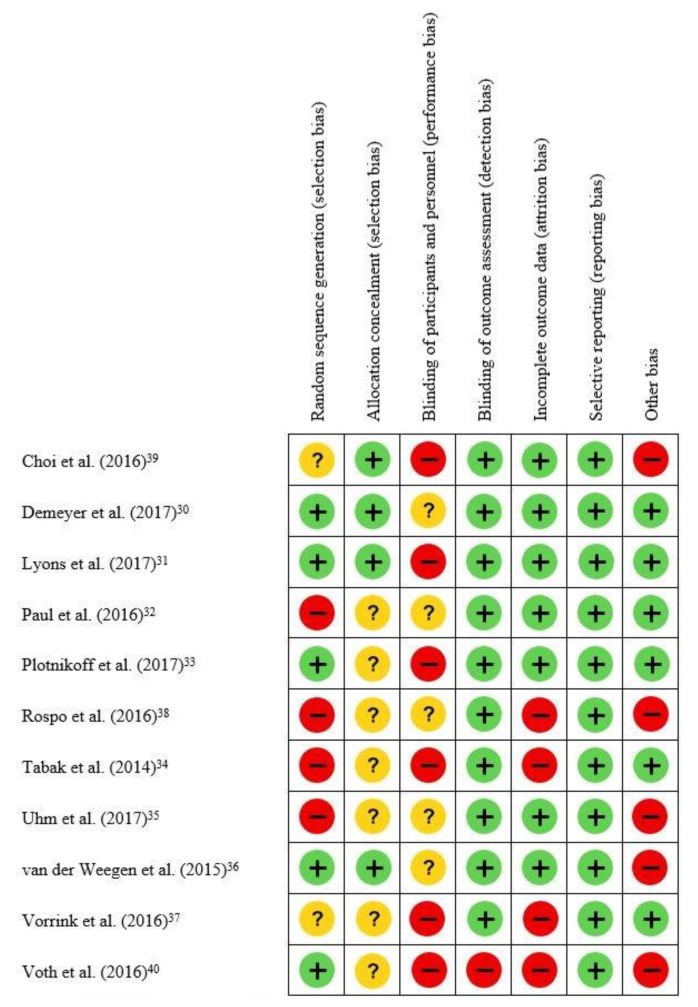
Risk of bias of the included studies (n=11). Legend: 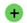
—Low risk of bias; 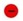
—high risk of bias; 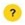
—Unclear risk of bias.

Agreement between the two reviewers was moderate, with a percentage of agreement of 71.4% and a Cohen’s Kappa of 0.50 (95% CI = 0.32; 0.68). None of the 11 included studies reported blinding of the participants, researchers, and assessors. Reporting bias (selective reporting of outcome) was not detected in any of the included studies and detection bias was only identified in one study [40]. Besides, in the 11 studies included, 8 studies were considered to present selection bias: five studies presented selection bias due to lack of randomization and allocation concealment [32,34,35,37,38], while the remaining three studies presented selection bias due to lack/inadequate allocation concealment only [33,40], or the randomization process [39]. Attrition bias was considered to be present in four studies, mainly due to the loss of a high number of participants during interventions compared to the initial sample size [34,37,38,40], Five included studies were judged as presenting bias from others sources, in particular not reporting on the validity and reliability of the applications used to measure physical activity [35,36,38,39,40] (Figure 2).

Agreement between the two reviewers regarding the assessment of methodological quality (PEDro scale) in the included studies was substantial, with a percentage of agreement of 88.2% and a Cohen’s Kappa of 0.76 (95% CI = 0.64; 0.88). The methodological quality of the included studies ranged between 2 [38] and 7 [31,36,39], out of a maximum score of 10. Of the 11 included studies, only 4 had a total of 6 or higher [30,31,36,39] (Table 1).

The quality of evidence varied between very low to low and is displayed in Table 2.

### 3.4. Primary Outcome: Physical activity

Physical activity was characterized by the number of steps [30,31,32,33,34,37,38,39], by the time spent in moderate to vigorous physical activity [36] and by self-report [35,40].

#### 3.4.1. Number of Steps

**Figure 3:**
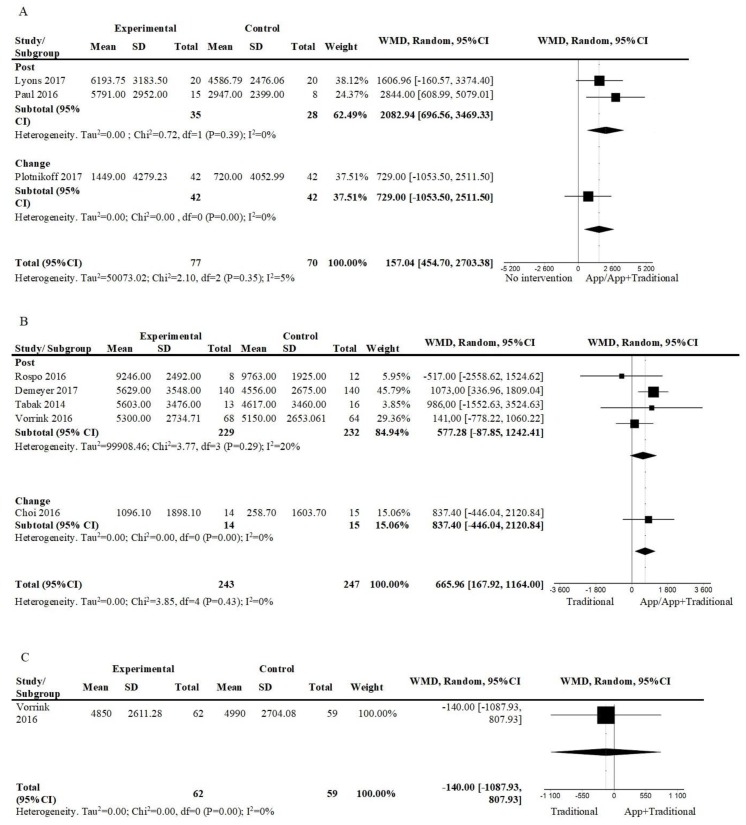
Forest plot of comparison, outcome: number of steps: (**A**) App/App+Traditional vs. No intervention, short term; (**B**) App/App+Traditional vs. Traditional, short term; (**C**) App+Traditional vs. Traditional, follow-up. Abbreviations: CI, confidence interval; df, degrees of freedom; WMD, weighted mean difference.

Figure 3A shows the pooled effects of an app-based intervention to promote physical activity alone [31,32], or combined with face-to-face interventions [33], against no intervention in the short term. The results suggested a positive effect, favouring the app-based interventions (3 studies; n = 147; WMD = 1579.04, 95%CI = 454.70, 2703.38; I^2^ = 5%). The overall quality of evidence was very low.

When comparing an intervention including a mobile app plus other interventions against other interventions in the short term (Figure 3B), results suggested a positive effect favoring the interventions, including a mobile app (5 studies; n = 490; WMD = 665.96; 95%CI = 167.92, 1164.00; I^2^ = 0%). The overall quality was low. Only one of these studies assessed the effect at follow up (24 weeks) and results show low-quality evidence that no intervention was superior (n = 121; WMD = −140, 95%CI = −1087.93, 807.93); (Figure 3C).

#### 3.4.2. Minutes in Moderate to Vigorous Physical Activity

**Figure 4:**
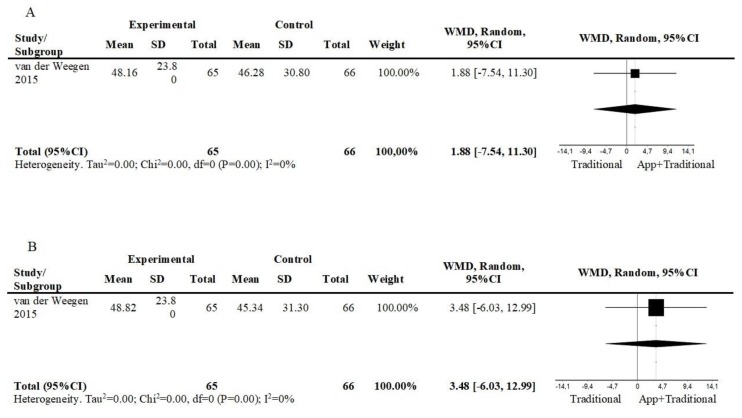
Forest plot of comparison, outcome: minutes in moderate to vigorous physical activity: (**A**) App+Traditional vs. Traditional, short term; (**B**) App+Traditional vs. Traditional, follow-up. Abbreviations: CI, confidence interval; df, degrees of freedom; WMD, weighted mean difference.

One study compared an intervention consisting of a mobile app, plus other interventions against a self-management plus usual care intervention. At short term, there was a small and non-significant effect in the group using the app at short term (1 study; n = 131; WMD = 1.88; 95%CI = −7.54, 11.30) (Figure 4A), and at follow up (12 weeks) (1 study; n = 131; WMD = 3.48; 95%CI = −6.03, 12.99) (Figure 4B).

#### 3.4.3. Self-Reported Physical Activity

**Figure 5:**
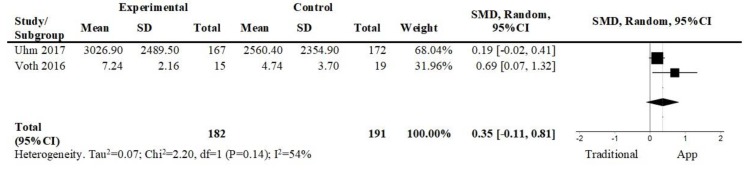
Forest plot of comparison. App vs. Traditional, short term, outcome: self-reported physical activity. Abbreviations: CI, confidence interval; df, degrees of freedom; SMD, standardized mean difference.

Figure 5 shows the pooled effect of two studies that compared app-based interventions against programs of exercise with pre-defined goals and there was a small and non-significant effect favoring the app-based intervention in the short term (2 studies; n = 373; SMD = 0.35; 95% CI = −0.11, 0.81; I^2^ = 54%). The overall quality of evidence was very low.

### 3.5. Secondary Outcomes

#### 3.5.1. Sedentarism

**Figure 6:**
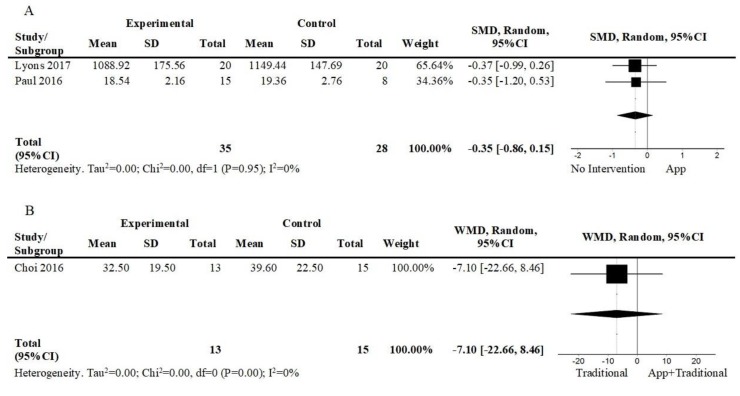
Forest plot of comparison, outcome: sedentarism: (**A**) App vs. No intervention, short term; (**B**) App+Traditional vs. Traditional, short term. Abbreviations: CI, confidence interval; df, degrees of freedom; WMD, weighted mean difference.

Three studies assessed sedentarism [31,32,39]. Figure 6A shows the pooled effects of mobile app-based interventions against no intervention in the short term [31,32]. There was a small and non-significant effect of the app-based interventions (2 studies; n = 63; SMD = −0.35; 95%CI = −0.86, 0.15; I^2^ = 0%). The overall quality of evidence was very low. One study [39] compared an intervention consisting of a mobile app plus other interventions against other interventions in the short term (Figure 6B). There was a small and non-significant effect favoring the intervention, including the mobile app (1 study; n = 28; WMD = −7.10; 95%CI = −22.66, 8.42). The overall quality of evidence was low.

#### 3.5.2. Self-efficacy in Exercise/Physical Activity

**Figure 7:**
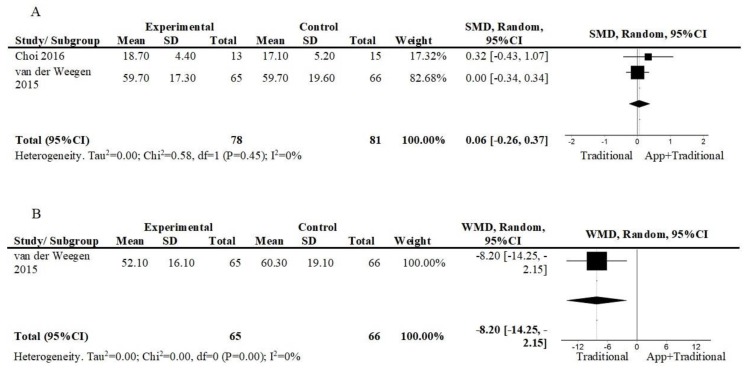
Forest plot of comparison, outcome: self-reported self-efficacy in exercise/physical activity: (**A**) App+Traditional vs. Traditional, short term. Abbreviations: CI, confidence interval; df, degrees of freedom; SMD, standardized mean difference; (**B**) App+Traditional vs. Traditional, follow-up. Abbreviations: CI, confidence interval; df, degrees of freedom; WMD, weighted mean difference.

Two studies [36,39] assessed the effect in the perceived self-efficacy during exercise or physical activity of an intervention consisting of a mobile app, plus other interventions against other interventions in the short term (Figure 7A). The results suggest a non-significant effect (2 studies; n = 159; SMD = 0.06; 95%CI = −0.26, 0.37; I^2^ = 0%). The overall quality of evidence was low. Moreover, van der Weegen et al. [36] also compared both interventions at follow-up (12 weeks) (Figure 7B). The results favoured usual care (1 study; n = 121; WMD = −8.20; 95%CI = −14.25, −2.15). The overall quality of evidence is low.

## 4. Discussion

Improving the physical activity levels of those that practice less than the recommended dose of physical activity is seen as of paramount importance, considering its general benefits across all age ranges, levels of care and pathologies. Arguments in favor of using mobile apps to promote physical activity include the possibility of reaching a high number of individuals at low costs [11,12]. This systematic review with meta-analysis’ results, based on very low to low-quality evidence, suggest a small and positive effect favoring interventions using a mobile app (both as a standalone treatment and when combined with other traditional interventions), in the short term both when compared with no intervention and other/traditional interventions and when number of steps is the outcome of interest, but not when time spent in physical activity or self-report were the outcomes of interest. Nevertheless, the results also suggested that interventions combining mobile apps and other/traditional interventions are not superior to other/traditional interventions for increasing the number of steps in the long term or decreasing sedentarism and are inferior to usual care for increasing physical activity/exercise self-efficacy in the long term. Considering that the evidence is of very low to low quality, there is a high degree of uncertainty around the estimates and further research is required and very likely to change both the degree of confidence in the estimate and the estimate itself [41].

The results of the present systematic review are encouraging when considering previous systematic reviews on the effectiveness of the information and communication technologies. A systematic review on the effectiveness of eHealth interventions for the promotion of physical activity in those aged 55 years or older concluded that eHealth interventions can effectively promote physical activity when compared to no intervention in the short term [42]. Another systematic review aiming to summarize the evidence about the effectiveness of web-based physical activity interventions in adults with chronic disease concluded that there is conflicting evidence on the effectiveness of web-based physical activity interventions in patients with a chronic disease [43]. Nevertheless, all these reviews are unanimous that further research is needed to investigate the effectiveness of information and communication technologies to promote physical activity and related variables.

As reported, this systematic review with meta-analysis’ results should be taken with caution, due to the low quality of the evidence. The quality tools used revealed that included studies fail to adequately report on allocation concealment, use small sample sizes, do not report on the reliability and validity of measurement instruments/apps, fail to report measures from at least 85% of subjects and fail to adjust for between-group differences at baseline. Overall, 7 out of 11 included studies were rated 5 or less in the PEDro scale, which is below the recommended cut off point of 6 for high-quality studies [23]. This led to the evidence being downgraded for study design and risk of bias and imprecision. Besides, studies showed high heterogeneity and varied in terms of duration of interventions, characteristics of the sample (e.g., asymptomatic vs. patients with a specific disease) and the use of co-interventions and, therefore, evidence had to be downgraded for inconsistency and indirectness [24,44]. Future studies should try to overcome these limitations to provide high-quality evidence.

Interestingly, most experimental groups did not receive a purely app-based intervention, rather the app was part of a package including face-to-face sessions [30,33,36,39], calls [31] and usual care [34], raising the question as to whether effectiveness may vary depending on whether the app is part of a package or is used as the only intervention. Furthermore, how the app is used (alone or in combination with other interventions) may vary depending on the characteristics of the population (e.g., for asymptomatic individuals the use of an app-based physical intervention may be appropriate, but for individuals with pathology an app-based intervention is likely to be part of a package of interventions in a biopsychosocial perspective that addresses the individual as a whole). Therefore, there is a need to fully describe the theory on which the design of the intervention is grounded and how exactly the app was used and framed within the intervention. Additionally, the characteristics of individuals more likely to respond positively to interventions using mobile apps need to be explored in future studies.

### Limitations

The studies included in this systematic review were limited in number, and of low quality. Therefore the confidence in its conclusions is limited. Additionally, we did not search grey literature and we did not include in our search strategy key words relating to specific sports or activities (e.g., cycling, stairs, flights climbed), so there might be missing publications that were not gathered by our search strategy. Due to the small number of studies we included in the same meta-analysis data from samples with different characteristics (e.g., asymptomatic and patients with different pathologies) and which, conceivably, can respond differently to app-based interventions. Further research is required to focus on randomized controlled studies of high quality that investigate the short and long-term effects of app-based interventions for different populations.

## 5. Conclusions

Very low to low-quality evidence suggests a small and positive effect, favoring interventions using mobile apps both as standalone interventions or combined with traditional interventions in the short term and when compared to no interventions or to traditional interventions and when the number of steps was the outcome of interest. Very low to low-quality evidence suggests that interventions using mobile apps are not superior to the traditional interventions for increasing the number of steps in the long term or decreasing sedentarism and increasing self-efficacy in the short and long term. Further high-quality studies are required and their results are likely to change both the estimates and the confidence in the results.

## Figures and Tables

**Table 1:** Quality assessment (PEDro scale) of the included studies (n = 11).

Author (Year)	1 *	2	3	4	5	6	7	8	9	10	11	Total Score
Choi et al. [39]	1	1	1	1	0	0	0	1	1	1	1	7/10
Demeyer et al. [30]	1	1	1	1	0	0	0	0	1	1	1	6/10
Lyons et al. [31]	1	1	1	1	0	0	0	1	1	1	1	7/10
Paul et al. [32]	1	0	0	1	0	0	0	1	1	1	1	5/10
Plotnikoff et al. [33]	1	1	0	0	0	0	1	0	1	1	1	5/10
Rospo et al. [38]	1	0	0	0	0	0	0	0	0	1	1	2/10
Tabak et al. [34]	1	1	0	0	0	0	0	0	1	1	1	4/10
Uhm et al. [35]	1	0	0	0	0	0	0	1	0	1	1	3/10
van der Weegen et al. [36]	1	1	1	1	0	0	0	1	1	1	1	7/10
Vorrink et al. [37]	1	1	0	1	0	0	1	0	0	1	1	5/10
Voth et al. [40]	1	1	0	1	0	0	0	0	0	1	1	4/10

Legend: 1: Yes, 0: No; Criteria: 1—Eligibility criteria; 2—Randomised allocation; 3—Concealed allocation; 4—Similar at baseline; 5—Blinded subjects; 6—Blinded therapists; 7—Blinded assessors; 8—Measures of at least one key outcome obtained for 85% of subjects; 9—Intention-to-treat analysis; 10—Between-group comparisons for at least one key outcome; 11—Point and variability measures for at least one key outcome; * Criteria 1 does not contribute for the total score in PEDro scale.

**Table 2:** Grading of Recommendations, Assessment, Development, and Evaluation (GRADE) evidence profile and summary of findings.

	Quality Assessment						Number of Participants		Effect Size	
Outcome	Studies (n)	Design	Risk of bias	Inconsistency	Indirectness	Imprecision	App/App+ traditional	No/traditional intervention	Absolute (95% CI)	Quality
Number of steps short term (app/app+traditional vs. no intervention)	3	2 RCT+ 1 Quasi-RCT	Serious *	Not serious	Serious †	Serious ‡	77	70	WMD 157.04 [454.70, 2703.38]	+ooo Very low
Number of steps short term (app/app+traditional vs. traditional)	5	4 RCT+ 1 Quasi-RCT	Serious *	Not serious	Serious †	Not serious	243	247	WMD 665.96 [167.92, 1164.00]	++oo Low
Number of steps follow-up (app+traditional vs. traditional)	1	RCT	Serious *	Not serious	Not serious	Serious ‡	62	59	WMD −140.00 [−1087.93, 807.93]	++oo Low
Minutes in MVPA short term	1	RCT	Not serious	Not serious	Serious †	Serious ‡	65	66	WMD 1.88 [−7.54, 11.30]	++oo Low
Minutes in MVPA follow-up	1	RCT	Not serious	Not serious	Serious †	Serious ‡	65	66	WMD 3.48 [−6.03, 12.99]	++oo Low
Self-reported PA short term	2	1 RCT+ 1 Quasi-RCT	Serious *	Serious §	Not serious	Serious ‡	182	191	SMD 0.35 [−0.11, 0.81]	+ooo Very low
Sedentarism short term (app vs. no intervention)	2	1 RCT+ 1 Quasi-RCT	Serious *	Not serious	Not serious	Serious ‡	35	28	SMD −0.35 [−0.86, 0.15]	+ooo Very low
Sedentarism short term (app+traditional vs. traditional)	1	RCT	Not serious	Not serious	Serious †	Serious ‡	13	15	WMD −7.10 [−22.66, 8.46]	++oo Low
Self-efficacy—short term	2	RCT	Not serious	Not serious	Serious †	Serious ‡	78	81	SMD 0.06 [−0.26, 0.37]	++oo Low
Self-efficacy—follow-up	1	RCT	Not serious	Not serious	Not serious	Serious ‡	65	66	WMD −8.20 [−14.25, 2.15]	++oo Low

* More than 25% of the studies were scored with high risk of bias (PEDro score lower than 6 and qualitative analysis of the risk of bias tool); † Heterogeneous samples and/or co-intervention in the experimental group; ‡ Total number of participants inferior to 400; § Heterogeneity was high with I2 superior to 50%. Abbreviations: RCT, randomized controlled trial; WMD, weighted mean difference; MVPA, moderate-vigorous physical activity; PA, physical activity; SMD, standardized mean difference.

## Supplementary Materials

The following are available online at https://www.mdpi.com/1660-4601/17/7/2251/s1, Supplementary table S1 Characteristics of the included studies (n = 11).
