# Supplementary material for: Effectiveness of Mobile Applications Running on Smartphones to Promote Physical Activity: A Systematic Review with Meta-Analysis
Source: Int J Environ Res Public Health. 2020 Mar 27;17(7):2251. doi: 10.3390/ijerph17072251 (PMC7177448; doi:10.3390/ijerph17072251)
Supplement: Supplementary file 1 [file ijerph-17-02251-s001.pdf]

Supplemental material S1 Characteristics of the included studies (n=11)

**Author (Year)**	**Study design**	**Name of mobile app**	**Participants**	**Control intervention**	**Experimental Intervention**	**Follow-up**	**Outcomes measures**
Choi et al. (2016)[39]	Pilot RCT	Not provided	**Control group:** n=15 sedentary pregnant women; Mean±SD: Age - 34.5±2.5 years; BMI - 27.4±3.9 kg/m2. **Experimental group:** n=15 sedentary pregnant women; Mean±SD: Age - 32.9±2.5 years; BMI - 28.0±3.7 kg/m2.	**Duration:** 12 weeks. **Initial in-person session (30 min):** Education plus instruction to increase number of steps to at least 8500 steps/day 5 days. **1-2 weeks:** Only Fitbit Ultra without access to the level of PA. **2-12 weeks:** Only Fitbit Ultra with access to PA parameters.	**Duration:** 12 weeks; **Initial in-person session (30 min):** Education plus instruction to increase number of steps to at least 8500 steps/day 5 days. **1-2 weeks:** Fitbit Ultra without access to the level of PA. **2-12 weeks:** Fitbit Ultra with access to PA parameters and mobile app (data inputted by participants); App sent daily messages (text or video) to support PA or to reinforce the topics on the initial in-person session, provided an activity diary, feedback about weekly step goal and tips about PA, diet and weight management.	No follow-up.	Primary outcome: Number of steps/day.<br><br>Secondary outcomes: Self-Efficacy for Physical Activity and time spent on TV/computer in hours/week.
Demeyer et al. (2017)[30]	Multicentre RCT	Fitbug app	**Control group:** n=172 (108 ♂) with COPD; Mean±SD: Age - 67±8 years; BMI - 25.9±4.8 kg/m2. **Experimental group:** n=171 (111 ♂) with COPD; Mean±SD: Age - 66±8 years; BMI - 26.7±5.3 kg/m2.	**Duration:** 12 weeks. Leaflet explaining the importance of PA in COPD and with recommendations for PA. One-to-one discussion (5-10 min.) with the investigator about the information in the leaflet. The usual medical treatment was not altered.	**Duration:** 12 weeks. Leaflet explaining the importance of PA in COPD and with recommendations for PA. One-to-one discussion (5-10 min.) with the investigator about the information in the leaflet and about motivation, barriers, favourite activities and strategies to become more active. The usual medical treatment was not altered. Leaflet with home exercises. Step counter (Fitbug Air) with feedback on the step count, a smartphone with Fitbug app and a project-tailored coaching app. App provided an activity goal (number of steps) and feedback on a daily basis. Patients' targets were automatically revised every week based on performance in the preceding week. Weekly text message with activity proposals sent by the investigators and telephone contacts (if needed).	No follow-up.	Primary outcome: Number of steps/day.

Lyons et al. (2017)[31]	Pilot RCT	Jawbone Up app	**Control group:** n=20 (3 ♂) overweighted or obese (grade 1); Mean±SD: Age - 61.70±6.26 years; BMI - 30.68±4.01 kg/m2.<br>**Experimental group**: n=20 (3 ♂) overweighted or obese (grade 1); Mean±SD: Age - 61.25±5.00 years; BMI - 30.00±2.86 kg/m2.	**Duration:** 12 weeks.<br>No intervention.	**Duration:** 12 weeks.<br>A mini tablet mobile device with the Jawbone Up app plus a wearable electronic activity monitor (Up24). App provided activity feedback, social support and the possibility to set goals. Also received counselling calls (15-20 min each) which included a check-in for any adverse events or technical problems, revaluation of weekly step goals, and action planning for the next week. Participants were asked to take at least 7000 steps/day on 2 days/week and to increase over time to at least 5 days/week. Sedentary bout goals were also negotiated with 1h being the number suggested.	No follow-up.	Primary outcome: Number of steps/day.<br><br>Secondary outcomes: Sitting time in minutes/day.
Paul et al. (2016)[32]	Quasi-RCT	STARFISH	**Control group:** n=8 (4 ♂) stroke survivors; Mean±SD: Age - 55.3±12.6 years; BMI - 24.8±1.8 kg/m2.<br>**Experimental group:** n=15 (8 ♂) stroke survivors; Mean±SD: Age - 56.3±8.7 years; BMI - 24.1±3.5 kg/m2.	**Duration**: 6 weeks.<br>No intervention.	**Duration:** 6 weeks.<br>Smartphone with the STARFISH app. App provided feedback for PA levels (participant own level and group members level), social support, individual and group rewards and the possibility to set goals. In week 1, the goal was mean number of steps/day recording during 1 week prior to the begging of intervention plus 10%. If the goal was achieved in 5 days in 1 week, it was increased by 5%.	No follow-up.	Primary outcome: Number of steps/day.<br><br>Secondary outcomes: Sedentary time in hours/day.

Plotnikoff (2017)[33]	RCT	eCoFit	**Control group:** n=42 (13 ♂) at risk of or diagnosed with diabetes type 2 or overweight/obese; Mean±SD: Age - 45.1±14.7 years; BMI - 31.7±5.1 kg/m2. **Experimental group:** n=42 (12 ♂) at risk of or diagnosed with diabetes type 2 or overweight/obese; Mean±SD: Age - 44.2±13.5 years; BMI - 35.0±5.9 kg/m2.	**Duration:** 20 weeks. No intervention.	**Duration:** 20 weeks. **1-10 weeks:** Five face-to-face group session (90 min.) comprised cognitive mentoring (30 min.) that aimed to educate participants about strategies to overcome barriers and increase motivation for and adherence to PA plus outdoor PA (60 min.) that aimed to provide participants with the confidence and skills to participate in sessions using the outdoor environment (e.g., benches, stairs) to increase muscular strength and aerobic fitness. Participants were also instructed to use the eCoFit app that included workout circuits around the city of Newcastle with challenges, instruction on how and where to use outdoor physical environment to be more physically active, a time tracer, a map of each location, visual instructions of exercises, an option to complete workout at home, goals setting, self-monitoring function and a link to social media (Facebook). **11-20 weeks:** Only eCoFit app.	No follow-up.	Primary outcomes: Number of steps/day.
Rospo et al. (2016)[38]	Quasi-RCT	Fitbit app;	**Control group:** n=12 (5 ♂) healthy; Mean±SD: Age - 45±3 years; BMI - 27.00±4.70 kg/m2. **Experimental group:** n=8 (3 ♂); Mean±SD: Age - 40±10 years; BMI - 23.70±3.53 kg/m2.	**Duration:** 2 weeks; Instructions to follow an intensity training based on the ACSM guidelines and attend training sessions 3/4 times per week. During the sessions, participants received personal feedback. Also asked to wear the HR monitors and step counters for the whole day during the entire intervention period without a specific goal.	**Duration:** 2 weeks; **Experimental group:** App connected to a pedometer that measured the number of steps/day. App allowed to assess to the progress. It did not provided strategy on how to achieve the goal neither gave reminders. The goal was to complete 10000 steps/day.	No follow-up.	Primary outcomes: Number of steps/day.

Tabak et al. (2014)[34]	Pilot RCT	Not provided	**Control group:** n=16 (11 ♂) with COPD who are not attending a physiotherapist; Mean±SD: Age - 67.9±5.7 years; BMI - 29.2±4.7 kg/m2. **Experimental group:** n=14 (8 ♂) with COPD who are not attending a physiotherapist; Mean±SD: Age - 65.2±9.0 years; BMI - 28.4±7.8 kg/m2.	**Duration:** 4 weeks; Usual care (for example, medication and physiotherapy).	**Duration:** 4 weeks. **1 week:** Baseline measurement. **2-4 week:** Usual care plus tele-rehabilitation intervention. The tele-rehabilitation intervention consisted of an activity coach (accelerometer plus a smartphone) for registration, feedback on PA and goal setting and a web portal with a symptom diary for self-treatment of exacerbations and an overview of the measured activity levels. In addiction, participants received feedback text messages. They were intruded to use the app for a minimum of four days per week.	No follow-up.	Primary outcomes: Number of steps/day.
Uhm et al (2017)[35]	Multicentre Quasi-RCT	Smart After Care	**Control group:** n=177 (0 ♂) with breast cancer that received treatment; Mean±SD: Age - 51.3±10.7 years; BMI - 23.3±3.3 kg/m2. **Experimental group:** n=179 (0 ♂) with breast cancer that received treatment; Mean±SD: Age - 49.3±8.0 years; BMI - 23.3±3.1 kg/m2.	**Duration:** 12 weeks. Leaflet with a program of aerobic and resistance exercises. Each participants had a goal for aerobic and resistance exercise. If at week 6, the participants accomplished their goals, new ones were defined for the next 6 weeks.	**Duration:** 12 weeks. InBodyBand pedometer plus the Smart After Care app. **1 week:** Baseline measurement of the PA level by the pedometer. **2-12 weeks:** A weekly goal was defined based on the usual minutes of physical activity and the number of minutes of prescribed aerobic exercise/week. App also allowed to watch a video clip of resistance and stretching exercises prescribed and to enter the number of sets for each resistance exercise performed. Mediation notice was displayed on the app only for patients receiving hormonal therapy.	No follow-up.	Primary outcome: International Physical Activity Questionnaire-Short Form (IPAQ-SF).

van der Weegen et al. (2015)[36]	Clustered RCT	It's LiFe!	**Control group:** n=66 (35 ♂) with COPD or diabetes type 2;<br>Mean±SD: Age - 56.9±8.3 years; BMI - 29.5±5.9 kg/m2.<br>**Experimental group:** n=65 (31 ♂) with COPD or diabetes type 2;<br>Mean±SD: Age - 57.5±7.0 years; BMI - 30.4±5.7 kg/m2.	**Duration:** 24 weeks**;**<br>Self-management support program plus usual care.<br>**1-2 week:** First session of the self-management program with a nurse. In the session was given information about the importance of PA, the risks of inactivity and a list of locally organized PA activities.<br>**3-26 week:** Three sessions of self-management program (3 week, 2-3 months and 4-6 months) with a nurse: second session: setting of a personal goal in minutes of activity/day and an activity plan; third session: information about activity results, barriers and facilitators, discussion of creation of new PA habits and reconsideration of the goals; fourth session: evaluation of the activity results, barriers, facilitators and PA habits.	**Duration: 24 weeks.**<br>Monitoring and feedback tool, self-management support program plus usual care.<br>**1-2 week:** Same of the control group.<br>**3-26 week:** Self-management program as described in the control group. Participants also used the monitoring and feedback tool (activity monitor, mobile phone app plus a web app).<br>Mobile and web app allowed to see real-time activity results and history in minutes of moderate to vigorous activity in relation to a personal goal defined according to participants baseline measurements. In addition, automated feedback messages were sent related to the personal goal. Activity results were visible for a nurse on the Web app.	12 weeks.	Primary outcome: Average minutes/day of moderate to vigorous PA.<br><br>Secondary outcomes: Exercise self-efficacy scale.

Vorrink et al. (2016)[37]	Multicentre RCT	Not provided	**Control group:** n=73 (36 ♂) with COPD; Mean±SD: Age - 63±8 years; BMI - 26.7±5.1 kg/m2. **Experimental group:** n=84 (42 ♂) with COPD; Mean±SD: Age - 62±9 years; BMI - 27.7±5.3 kg/m2.	**Duration:** 24 weeks. Usual care.	**Duration:** 24 weeks. Smartphone app plus website for the physiotherapists. App allowed to see real time measurement of PA and to set a goal for PA. In the first week, the app measured PA level in order to set a goal for the next weeks. The goal was defined according to: (i) average steps/day plus 20% as daily step goal; (ii) daily, the number of steps during the 30 most intensive minutes were counted and averaged into a value for a week; and (iii) 30 intensive minutes performed per day, according to the Dutch healthy exercise norm. Through the website, physiotherapists could reduce or increase the amount and intensity of the physical activity goal, see detailed information about participants and send group or individual text messages.	24 weeks.	Primary outcome: Number of steps/day.
Voth et al. (2016)[40]	Pilot RCT	Not provided	**Control group:** n=28 (12 ♂); Mean±SD: Age - 41.53±10.90 years; BMI - 25.87±3.60 kg/m2. **Experimental group:** n=28 (12 ♂); Mean±SD: Age - 37.45±14.13 years; BMI - 28.24±6.50 kg/m2.	**Duration:** 8 weeks. Definition of a personal goal for weekly exercise frequency. Participants were encouraged to implement their goals and did not receive any additional support throughout the duration of the study.	**Duration:** 8 weeks. Assess to an app that allowed daily monitoring of exercise behaviour and setting of a personal goal for the frequency of exercise. The goal was defined in the begging of the intervention. Additional, text messages were sent when participant did not used the app and to delivered information in the beginning of each week about the components of self-monitoring, verbal persuasion, performance accomplishment and vicarious experience.	No follow-up.	Primary outcome: Godin Leisure Time Exercise Questionnaire (GLTEQ).

Abbreviations: RCT: randomized controlled trial; ♂: male; SD: standard deviation; COPD: chronic obstructive pulmonary disease; kg/m2: kilograms per meter squared; BMI: body mass index; min: minutes; PA: physical activity; app: mobile application.
